# COVID-19 Related Fear, Risk Perceptions, and Behavioral Changes According to Level of Depression among Nursing Students

**DOI:** 10.3390/ijerph19084814

**Published:** 2022-04-15

**Authors:** Kyungmi Kim, Hyesun Jeong, Jongeun Lee

**Affiliations:** 1 Department of Nursing, Gangdong University, Eumseong-gun 27690, Korea; 96yasi@gangdong.ac.kr; 2 Department of Nursing, College of Nursing & Health, Kongju National University, Gongju-si 32588, Korea; hsjeong@kongju.ac.kr; 3 Department of Nursing Science, College of Medicine, Chungbuk National University, Cheongju-si 28644, Korea

**Keywords:** COVID-19, nursing students, depression, fear, risk perception, behavior

## Abstract

Depression may have a negative impact on health behaviors during crisis situations, such as the COVID-19 pandemic. Accordingly, the present study aimed to investigate the effects of depression felt by nursing students on their infectious disease response. A total of 241 nursing students from two nursing colleges in Chungcheong Province was convenience sampled between 2 and 12 December 2020. The tools used in the study were the Patient Health Questionnaire-9 Korean version, Fear of COVID-19 Scale, COVID-19-related risk perceptions, and COVID-19 behavior changes. The depression group showed higher fear scores and lower behavioral change scores than the non-depression group. Such findings indicated that the depression group did not actively perform COVID-19-related preventive behaviors. With respect to the influencing factors of depression, depression scores were 2.28 times higher among sophomores than seniors; fear scores were 1.09 times higher in the depression group than the non-depression group; and behavioral change scores were 0.87 times lower in the depression group than the non-depression group. Based on the findings in the present study, it is necessary to screen nursing students with depression during disaster crisis situations, such as the COVID-19 pandemic, and provide active psychological support to such students for their mental health care.

## 1. Introduction

As the COVID-19 pandemic has continued to spread throughout the world, leading to an increasing number of mass outbreaks and deaths [1], changes in daily life have continued during the “with corona” situation, indicating living with the disease. According to Worldometers, the global cumulative confirmed cases of COVID-19 was over 401.18 million cases (as of 9 February 2022) [2]. Such increases in confirmed cases are appearing due to the relaxation of quarantine rules as a result of the wide availability of COVID-19 vaccine, rapid spread of infections by the Delta variant, and breakthrough infections among individuals who have been vaccinated. 

The average age of the confirmed patients was 43.9 years, with individuals aged 20–59 years accounting for 69%. In particular, infection among individuals aged 20–29 years was 25.1%, which was higher than in any other age group [3]. A period such as a pandemic is when people must fight against an unknown disease, and thus, it can be viewed as an infection disaster situation [4], and as such a disaster lingers, it can have a negative impact on the mental health of each individual. Accordingly, people must be prepared for a mental-demic, where mental trauma spreads like an infectious disease [5]. Depression or lethargy from facing major changes in daily life due to the spread of COVID-19 is referred to as the “Corona Blues” [6]. According to the 2021 COVID-19 National Mental Health Survey, the number of individuals who experienced depression has increased by more than twice as compared to the results from the 2018 Community Health Survey, and in particular, individuals aged 20–39 years have shown the highest depression score [7].

Since the outbreak of COVID-19, the prevalence of depression has increased to 25%, representing an approximately seven-fold increase as compared to the global prevalence of depression of 3.44% in 2017, indicating that an increasing number of individuals are experiencing depression due to COVID-19 [8,9]. Such results suggest that COVID-19 is having a significant impact on the mental health of individuals. Social distancing due to COVID-19 has been reported to cause an increase in negative emotions due to various factors, including reduced close communication, fear of infection, inadequate information, and poor diet [10,11].

For nursing students, the COVID-19 pandemic has caused them to experience fear of infection, difficulties with clinical practical training, severed interpersonal relationships, and fear about academics and employment [12]. In particular, clinical practice for nursing students is a required curriculum, but nursing students have expressed that “Clinical practical training is still something that I am afraid of” [13]. Nursing students who went through the Middle East respiratory syndrome (MERS) outbreak in 2015 and the current COVID-19 pandemic were reported to experience fear of potential infection by the novel infectious disease and related emotional distress [12,14]. Such fear was found to have a negative impact on the quality of life of these nursing students, as well as their education and passing the nurse licensing examination [15]. Moreover, nursing students also experienced loss of confidence and depression due to such fear [12]. Accordingly, there is the need to systematically manage fear among nursing students, who will become healthcare professionals in the future [16].

Risk perception has been reported to be associated with health protective behaviors such as mask wearing or social distancing by influencing preventive measures through increased fear and anxiety [17]. Individuals with high-risk perception of infection tend to choose healthy behavior, which ultimately reduces the likelihood of infection [18]. However, because risk perception can also have a negative impact, such as depression, on people facing a public health crisis, psychological support must be considered [19]. Moreover, college/university students are one of the most dynamic groups with strong mobility and socialization, while they are also young, healthy, and often show only mild symptoms after being infected. Therefore, close attention is needed on risk perception. In particular, students in healthcare-related fields have a high risk of becoming a source of infection from coming into close contact with infected patients, and thus interest in risk perception is warranted [20].

Major COVID-19 prevention strategies implemented in 2020 included behavioral regulations, such as proper hand washing, social distancing, and mask wearing [21], emphasizing continued behavior changes for minimizing the impact of the COVID-19 pandemic [22]. Nursing students have become active actors who strictly abide by personal hygiene rules, such as mask wearing [23]. In addition, prolongation of the COVID-19 pandemic also appears to have had a significant influence on the lifestyle habits of young adults, including increased food intake, increased mental stress, and decreased alcohol intake. Therefore, prolongation of the COVID-19 pandemic can cause various mental health issues, including the Corona Blues, which requires appropriate preparedness [24].

Accordingly, considering that negative emotions (fear and depression) among nursing students due to their risk perception of COVID-19 may contribute to maladaptive health behaviors, the present study aimed to identify the differences in COVID-19 related fear, risk perception, and behavioral changes according to level of depression among nursing students and also to identify the influencing factors of depression among nursing students to provide basic data for establishing measures that can enhance infectious disease response among nursing students.

The primary objective of the present study was to identify the differences in COVID-19 related fear, risk perceptions, and behavioral changes according to level of depression among nursing students. The specific objectives were as follows:

(1) Identify differences in general characteristics according to level of depression among nursing students.
(2) Compare COVID-19 related fear, risk perceptions, and behavioral changes according to level of depression among nursing students.
(3) Identify the influencing factors of depression among nursing students.

## 2. Materials and Methods

### 2.1. Study Design and Participants

The present study used a descriptive survey design to identify the differences in COVID-19 related fear, risk perceptions, and behavioral changes according to level of depression among nursing students. The study population consisted of 241 nursing students (68, 71, and 102 sophomores, juniors, and seniors, respectively) from two nursing colleges in Chungcheong Province, Korea.

### 2.2. Research Instruments

#### 2.2.1. Patient Health Questionnaire-9 (PHQ-9) Korean Version

Level of depression was measured using the Korean version of PHQ-9, which was originally developed by Spitzer et al. [25] and subsequently adapted into Korean by Park et al. [26]. This questionnaire consisted of a total of nine items, with each item rated on a 4-point Likert scale (0: “not at all” to 3: “almost every day”). In the present study, students with a score of <5 points were assigned to the non-depression group, and those with a score of ≥5 points were assigned to the depression group [27]. The internal reliability index of the instrument was 0.88 in the study by Park et al. [26] and 0.80 in the present study.

#### 2.2.2. Fear of COVID-19 Scale (FCV-19S)

To measure fear of COVID-19, FCV-19, developed by Ahorsu et al. [28] and subsequently validated was translated by the researcher of the present study, and the content validity of the translated version was tested by one psychiatric nursing professor, one mental health nurse, and one advanced practice nurse in infection control. This instrument consisted of a total of seven items. Each item was rated on a 5-point Likert scale (1: “strongly disagree”, 2: “disagree”, 3: “neutral”, 4: “agree”, and 5: “strongly agree”) with higher total score indicating higher fear of COVID-19. The internal reliability index of the instrument was 0.82 in the study by Ahorsu et al. [28] and 0.84 in the present study.

#### 2.2.3. COVID-19 Related Risk Perceptions

To measure COVID-19 related risk perceptions, the COVID-19-related Risk Perceptions developed by Olapegba et al. [29] was translated by the researcher of the present study, and the content validity of the translated version was tested by one psychiatric nursing professor, one mental health nurse, and one advanced practice nurse in infection control. This instrument consisted of a total of nine items. Each item was rated on a 6-point Likert scale (1: “not at all”, 2: “almost never”, 3: “slightly”, 4: “not sure”, 5: “very much”, and 6: “extremely”) with high total score indicating higher COVID-19 related risk perceptions. The internal reliability index of the instrument was 0.76 in the study by Olapegabu et al. [29] and 0.85 in the present study.

#### 2.2.4. COVID-19 Behavior Changes

To measure behaviors that can help reduce the risk of COVID-19 infection, 12 behaviors developed by Barber and Kim [30] were translated into Korean, and the content validity of the translated version was tested by one psychiatric nursing professor, one mental health nurse, and one advanced practice nurse in infection control. The items consisted of: (1) washing hands more frequently, (2) taking more care about cleanliness, (3) wore a surgical/hygiene mask, (4) no shaking hands, (5) no face touching, (6) no socializing with others, (7) avoidance of public places (such as restaurants, public transportation, libraries, or stores), (8) complete quarantine, (9) more balanced diet in an effort to avoid contracting COVID-19, (10) taking additional vitamins or supplements in an effort to avoid contracting COVID-19, (11) purchasing extra food, and (12) purchasing extra medical supplies, including medications. For each item, the participants were instructed to choose from one of three choices—“Yes”, “I am considering it, but not yet doing it”, and “No”. In the subsequent analysis, the responses were converted to binary variables, with 1 point for “practice” and 0 points for the other two choices. The internal reliability index of the instrument was 0.81 in the study by Barber and Kim [30] and 0.73 in the present study.

### 2.3. Data Collection and Ethical Consideration

The present study was conducted with an approval from the Institutional Review Board of “C” University. Data were collected between 2 and 12 December 2020, using Google Forms as a non-face-to-face mobile questionnaire survey in consideration of preventing the spread of COVID-19. Recruitment announcement containing information regarding the objectives and goals of the study posted on SNS platforms to students in two colleges and a link (URL) to the Google Form containing an online consent form was provided. Upon collection of data from the target number of participants to be included in the final analysis, the link to the Google Form was closed. The questionnaire required approximately 10 min to complete. Mobile beverage coupons were issued as a small token of appreciation for participation to those who had provided their personal contact information after completing the survey. After the coupons were issued, all such personal information was destroyed.

### 2.4. Data Analysis

Data collected in the study were analyzed using the IBM SPSS Statistics 26.0 program. The general characteristics of the depression and non-depression groups were analyzed using frequency/percentage and mean/standard deviation (SD). The differences between the two groups according to their general characteristics were analyzed using χ^2^-test and Mann–Whitney test. Additionally, the differences in COVID-19 related fear, risk perceptions, and behavioral changes between the two groups were analyzed using independent t-test. Behavioral changes in the two groups were expressed as frequency and percentage. Moreover, binary logistic regression analysis was used to analyze the influencing factors of the depression group.

## 3. Results

### 3.1. General Characteristics of the Depression and Non-Depression Groups

The study population consisted of 241 nursing students, including 147 in the non-depression group (61.0%) and 94 in the depression group (39.0%). In the comparison by grade or year in school, the highest percentage of students in the depression group was sophomores (37.2%), followed in order by seniors (35.1%) and juniors (27.7%). There was a significant difference in the distribution by year in school between the depression and non-depression groups (x^2^ = 6.51, *p* = 0.04). With respect to gender, 87.2% were females, and 12.8% were males in the depression group, while there was no significant difference between the depression and non-depression groups. The two groups showed no difference in the distribution of religion, while both groups had over 50% of the students living alone or in a boarding house. Both groups reported that they received information about COVID-19 mostly through mass media, while over 90% in both depression and non-depression groups reported that they were doing “well” with respect to practicing preventive measures (Table 1).

### 3.2. COVID-19 Related Fear, Risk Perceptions, and Behavioral Changes in the Depression and Non-Depression Groups

Analysis of differences in COVID-19 related fear, risk perceptions, and behavioral changes between the depression and non-depression groups showed that there were significant differences in fear (t = −2.52, *p* = 0.012) and behavioral changes (t = 2.17, *p* = 0.031) between the two groups. Fear score was higher in the depression group (18.35 ± 4.84 points) than in the non-depression group (16.73 ± 4.90 points), whereas behavioral change score was higher in the non-depression group (6.64 ± 2.30 points) than in the depression group (5.96 ± 2.42 points; Table 2).

### 3.3. Differences in Behavioral Change Scores between the Depression and Non-Depression Groups

In the comparison of behavioral change items between the depression and non-depression groups, there were significant differences between the two groups with respect to “washed hands more frequently” (χ^2^ = 4.04, *p* = 0.04), “taken more care about cleanliness” (χ^2^ = 5.07, *p* = 0.02), “stopped touching own face” (χ^2^ = 6.15, *p* = 0.01), “eaten a more balanced diet in an effort to avoid contracting COVID-19” (χ^2^ = 4.23, *p* = 0.04), and “taken additional vitamins or supplements in an effort to avoid contracting COVID-19” (χ^2^ = 6.18, *p* = 0.01). Generally, the non-depression group showed higher behavioral change scores than the depression group (Table 3).

### 3.4. Associations between Depression and Fear and Behavioral Changes

A binary logistic regression analysis was performed on the non-depression group (PHQ-9 < 5) and depression group (PHQ-9 ≥ 5) to identify the influencing factors of depression among nursing students. Variables that showed significant differences between the depression and non-depression groups in the univariate analysis (grade, fear, and behavioral changes) were included as explanatory variables.

The analysis results showed that the variables associated with the depression group were being a sophomore (odds ratio [OR] = 2.28, *p* = 0.013, 95% confidence interval [CI]: 1.19–4.27), fear (OR = 1.09, *p* = 0.005, 95% CI: 1.03–1.15), and behavioral changes (OR = 0.87, *p* = 0.016, 95% CI: 0.77–0.97). In the depression group, sophomores were 2.28 times more depressed than the fourth years. Moreover, as fear score increased by 1 point in the non-depression group, the score increased by 1.09 times in the depression group, and as behavioral change score decreased by 1 point in the non-depression group, the score decreased by 0.87 times in the depression group. Finally, the probability of “no face touching” preventive behavior (OR = 0.48, *p* = 0.031, 95% CI: 0.24–0.93) was 0.48 times lower in the depressed group than in the non-depressed group, and “additional purchase of medical supplies” (OR = 3.13, *p* = 0.021, 95% CI: 1.19–8.22) was 3.13 times higher in the depressed group (Table 4).

## 4. Discussion

The present study aimed to identify the influence of level of depression among nursing students on their COVID-19 related fear, risk perceptions, and behavioral changes in order to establish basic data for responding to mental health issues faced by nursing students in the “with corona” era.

In this study, 94 students (39.0%) of all participants were classified as depressed. The depression experience rate of the subjects of this study was lower than the 59.9% reported in a previous study [31]. Of the 94 students, 35 (37.2%) sophomores without clinical experience and 33 (35.1%) fourth-year students who were about to find a job had higher rates of feeling depressed than the third-year students. Infected patients, self-quarantined persons, healthcare workers, and nursing students are vulnerable groups to depressive symptoms when an epidemic occurs [32], and thus, there is a need for active intervention and effective planning for them.

In the present study, the mean fear score was 18.35 ± 4.84 points in the depression group, which was significantly higher than 16.73 ± 4.90 points in the non-depression group. Another study that investigated COVID-19 related fear among nurses [33] reported a mean fear score of 19.92 points, indicating that nurses felt a higher level of fear than nursing students. A study on college students and graduates in Russia and Belarus [34] reported a mean fear score of 17.2 points, and the depression group in the present study showed a higher fear score. Such findings were consistent with other studies reporting that fear increased when depression was one of the personal characteristics that influenced COVID-19 related fear [35], and that students in healthcare-related majors had higher COVID-19 related fear than students in other majors [36]. Since fear of a novel infectious disease can cause an increase in the level of depression among individuals, enhancement of psychological health and social resilience for coping well with such fear is deemed necessary.

Although it is not a study on the relationship between COVID-19 related risk perception and depression, Park et al. [37] reported that high anxiety increases risk perception. However, the findings in the present study showed no difference in risk perception between the depression and non-depression groups. It is believed that there was no difference in risk perception between the two groups because both groups perceived the seriousness of COVID-19 infection through the pandemic situation and mass outbreaks in Korea [38]. Moreover, it can also be assumed that there was no difference between the two groups in the present study because both groups received their COVID-19 related information through mass media based on the fact that COVID-19 related risk perceptions increase when people obtain more information [31]. Therefore, health risks may increase with increasing level of fear or depression from exposure to false information through the media [39], and thus it is necessary to have a proper level of risk perceptions based on accurate information.

In the present study, the mean behavioral change score was significantly higher in the non-depression group (6.64 ± 2.30 points) than in the depression group (5.96 ± 2.42 points). Brouwer et al. [40] found that students who reported higher scores on self-care practices had lower psychological distress scores. Such findings indicated that the depression group experiencing psychological pain was less active in changing their behavior than the non-depression group, and that the depression group may take fewer preventive actions during the COVID-19 pandemic [41]. Therefore, closer attention should be paid to management of the depression group. Among the behavioral changes, the items for which more than 80% of nursing students responded that they practice were “washing hands more frequently”, “taking more care about cleanliness”, and “mask wearing”. In comparison to this, a study on COVID-19 related behavioral changes among the elderly and young adults [30] reported that over 80% of the participants practiced frequent hand washing, more care about cleanliness, stopped shaking hands, and avoidance of public places. These results showed that depressed nursing students were less sensitive to behavioral changes as compared to the elderly and young adults. Generally, lower level of fear is associated with less behavioral changes [29], but individuals suffering from psychological disorders, such as depression, have difficulty practicing proper adaptive behaviors. Accordingly, the depression group in the present study showed an increased level of fear but a decrease in behavioral changes. The findings in the present study confirmed the claims that the number of young people with depression is increasing rapidly in Korea [7] and that they may suffer from psychological distress, such as fear and depression, during the COVID-19 pandemic [42].

The influencing factors of depression identified in the present study were grade in school, fear, and behavioral changes. In this study, compared to the fourth years, the sophomores’ depression was 2.28 times higher, and the third year was 1.1 times higher. In the second year, the burden of major classes increases, and the fear of infection increases because there is no clinical field experience [43]. In addition, as activities are restricted due to social distancing, it is predicted that the likelihood of experiencing negative depression is higher [44]. It is believed that this is due to younger people with broader radii of activity in daily life being more likely to be affected by depression during the COVID-19 pandemic [45]. Nguyen et al. [46] also reported that senior nursing students have lower fear than low-grade nursing students, suggesting that their fears of COVID-19 are lower because seniors are more familiar with the infectious disease and preventive measures. In other words, as social adaptability improves as they get older, more attention is needed on depression in lower grades. Fear score was 1.09 times higher in the depression group than in the non-depression group. Such findings were consistent with the results showing that the group with fear had a significantly higher probability of belonging to the depression group [47]. Therefore, these findings indicated that it is necessary to understand that fear of a novel infectious disease can cause an increase in the level of depression among individuals [48] and provide appropriate psychological support during situations that are difficult to control by individuals.

The results showing that behavioral changes in the depression group were 0.87 times less than that of the non-depression group were contradictory to other results showing an increase in COVID-19 prevention practice, such as mask wearing and hand washing [38]. In the depressed group, the behavior of not touching their own face was 0.48 times lower than in the non-depressed group, and the behavior of buying extra medical supplies or drugs was 3.13 times higher than in the non-depressed group. The depressed group had low psychomotor activation, which led it to be less active and to have low expectations for enjoyment, resulting in a decrease in interest and pleasure [49]. It is believed that lethargy and despair resulting from continued social distancing due to prolongation of COVID-19 may have influenced depression [38]. Persistence of such states affects depression, and thus it is important to operate programs that people with depression can participate in and strengthen psychological support systems that promote participation in such activities during the COVID-19 pandemic.

The limitations in the present study include the fact that the study population consisted of nursing students from two colleges in Chungcheong Province. Therefore, the investigation and analysis results should be interpreted with caution when generalizing the findings for all nursing students. Despite this, the present study was significant in that it compared fear, risk perceptions, and behavioral changes among nursing students during the COVID-19 pandemic by dividing them into the depression and non-depression groups.

## 5. Conclusions

The present study is significant in that it identified COVID-19 related fear, risk perceptions, and behavioral changes according to level of depression among nursing students to establish basic data for responding to mental health issues of nursing students.

The findings in the present study showed that the depression group had a higher fear score than the non-depression group, but the depression group was less active in practicing preventive behaviors. In particular, in items such as frequent hand washing, maintaining cleanliness, no face touching, more balanced diet, and taking additional vitamins, the non-depressed group showed more healthy behaviors than the depressed group. On the other hand, compared to the non-depressed group, the depressed group did less no face touching and purchased more additional medical supplies. Therefore, it is necessary to take interest in the mental health care of depressed nursing students and provide them with active psychological support. Moreover, it is also necessary to find measures for increasing participation by lower grade students when providing psychological support. It is necessary to provide accurate information to reduce fear in the depression group and guide them about how exposure to too much information via SNS can further increase depression. The findings in the present study showed no difference in risk perceptions between the depression and non-depression groups due to the characteristics of participants being young students who actively acquire COVID-19 related information mostly through mass media. However, caution is needed since exposure to false information through mass media could cause increased fear or depression, which could lead to maladaptive health behavior. Finally, it is necessary to conduct replication studies on fear, risk perceptions, and behavioral changes in depressed groups to predict fear, risk perceptions, and behavioral changes in depressed groups during a novel infectious disease outbreak and respond appropriately.

## Figures and Tables

**Table 1:** General characteristics of the participants.

Variables	Categories	Total	Non Depression	Depression	χ^2^ or Z	*p*
		*n* (%)				
		241 (100.0)	147 (61.0)	94 (39.0)		
Grade	Sophomore	68 (28.2)	33 (22.4)	35 (37.2)	6.51	0.04
	Junior	71 (29.5)	46 (30.6)	26 (27.7)		
	Senior	102 (42.3)	69 (46.9)	33 (35.1)		
Gender	Male	34 (14.1)	22 (15.0)	12 (12.8)	0.23	0.63
	Female	207 (85.9)	125 (85.0)	82 (87.2)		
Religion	No	163 (67.6)	101 (68.7)	62 (66.0)	0.20	0.67
	Yes	78 (32.4)	46 (31.3)	32 (34.0)		
Type of residence	Living with parents	73 (30.3)	49 (33.3)	24 (25.5)	2.54	0.28
	Dorm	35 (14.5)	18 (12.2)	17 (18.1)		
	Live alone/boarding house	133 (55.2)	80 (54.4)	53 (56.4)		
COVID-19 information	University	10 (4.1)	6 (4.1)	4 (4.3)	0.06	0.96 *
resource	Mass media	231 (95.9)	140 (95.9)	90 (95.7)		
Observe precautions	Well Moderately	222 (92.4) 19 (7.6)	134 (91.2) 13 (8.8)	88 (93.6) 6 (6.4)	−0.69	0.49 *

* Mann–Whitney test.

**Table 2:** Comparison of fear, risk perceptions, and behavioral changes between the two groups.

Variables	Non Depression	Depression	t (*p*)
	Mean ± SD	Mean ± SD	
Fear	16.73 ± 4.90	18.35 ± 4.84	−2.52 (0.012)
Risk perceptions	33.70 ± 7.00	34.65 ± 6.48	−1.06 (0.292)
Behavioral changes	6.64 ± 2.30	5.96 ± 2.42	2.17 (0.031)

**Table 3:** Differences in behavioral changes between the two groups.

	Item		Non Depression (%)	Depression (%)	χ^2^	*p*
1	Wash hands more	Do	94.6	87.2	4.04	0.04
		Do not do	5.4	12.8		
2	More careful about cleanliness	Do	94.6	86.2	5.07	0.02
		Do not do	5.4	13.8		
3	Wore a mask	Do	100.0	100.0		
		Do not do	0.0	0.0		
4	Stop shaking hands	Do	65.3	53.2	3.52	0.06
		Do not do	34.7	46.8		
5	Stop touching own face	Do	38.8	23.4	6.15	0.01
		Do not do	61.2	76.6		
6	Stop socializing	Do	58.5	55.3	0.24	0.63
		Do not do	41.5	44.7		
7	Avoid public places	Do	65.3	62.8	0.16	0.69
		Do not do	34.7	37.2		
8	Gone into complete quarantine	Do	21.1	19.4	0.11	0.75
		Do not do	78.9	80.6		
9	Purchased extra food	Do	12.9	16.0	0.44	0.51
		Do not do	87.1	84.0		
10	Purchased extra medical supplies or medications	Do	12,9	22.3	3.67	0.06
		Do not do	87.1	77.7		
11	Eaten a more balanced diet	Do	49.7	36.2	4.23	0.04
		Do not do	50.3	63.8		
12	Taken additional vitamins	Do	50.3	34.0	6.18	0.01
		Do not do	49.7	66.0		

**Table 4:** Associations of grade, fear, and behavioral changes with the depressed group.

Variables	OR (95% CI)	*p*-Value
Grade		
Sophomore	2.28 (1.19–4.27)	0.013
Junior	1.10 (0.57–2.12)	0.778
Senior	1	
Fear	1.09 (1.03–1.15)	0.005
Behavioral Changes	0.87 (0.77–0.97)	0.016
No face touching	0.48 (0.24–0.93)	0.031
Purchasing extra medical supplies	3.13 (1.19–8.22)	0.021

OR = odds ratio; CI = confidence interval.

## Data Availability

The data presented in this study are available upon request from the corresponding author.
